# Topical Corticosteroid Misuse and Awareness of Steroid-Induced Dermatoses Among Adults in Saudi Arabia: A Cross-Sectional Survey

**DOI:** 10.7759/cureus.111499

**Published:** 2026-06-25

**Authors:** Ahmed Y Bukannan, Deema S Al Abdrabbuh

**Affiliations:** 1 Dermatology, Royal Medical Services, Riffa, BHR; 2 Dermatology, Royal Medical Services, Busaiteen, BHR

**Keywords:** adverse effects, corticosteroid misuse, cross-sectional survey, dermatology, knowledge and practices, public awareness, saudi arabia, social media influence, steroid-induced dermatoses, topical corticosteroids

## Abstract

Background: Topical corticosteroids are among the most commonly used medications in dermatology and play a pivotal role in the management of inflammatory skin diseases. However, inappropriate use, unsupervised access, and cosmetic misuse may lead to significant adverse effects and steroid-induced dermatoses. Data regarding public awareness, knowledge, and misuse of topical corticosteroids in Saudi Arabia remain limited.

Methods: A national cross-sectional survey was conducted among adults residing in Saudi Arabia between January and March 2026. Data were collected using a structured, self-administered online questionnaire assessing demographic characteristics, topical corticosteroid use, knowledge, awareness of steroid-induced dermatoses, adverse effects, and misuse practices. Topical corticosteroid misuse was defined as the presence of at least one inappropriate behavior, including non-prescription use, cosmetic use, social media-driven use, medication sharing, prolonged unsupervised use, reuse of previous prescriptions, or use for fungal infections. Multivariable logistic regression analysis was performed to identify independent predictors of misuse.

Results: A total of 1,248 participants were included in the analysis, of whom 736 (59.0%) were female. Previous topical corticosteroid use was reported by 842 participants (67.5%). Among users, 289 (34.3%) obtained topical corticosteroids without a prescription, 264 (31.4%) purchased them directly from pharmacies without a prescription, and 178 (21.1%) reported cosmetic use. Overall, 362 participants (29.0%) met the definition of topical corticosteroid misuse. Knowledge regarding corticosteroid safety was generally favorable, with 971 participants (77.8%) recognizing skin thinning as a potential adverse effect and 1,112 (89.1%) acknowledging the importance of medical supervision during prolonged use. Nevertheless, awareness of hidden corticosteroids in cosmetic products was lower (693 participants, 55.5%). Among corticosteroid users, 247 (29.3%) reported at least one adverse effect, most commonly acneiform eruptions (164 users, 19.5%). Social media recommendation was the strongest independent predictor of misuse (adjusted odds ratio (aOR): 3.48; 95% confidence interval (CI): 2.51-4.82; P<0.001), followed by facial application (aOR: 1.89; 95% CI: 1.40-2.55; P<0.001), female sex (aOR: 1.72; 95% CI: 1.29-2.31; P<0.001), and non-healthcare occupation (aOR: 1.64; 95% CI: 1.08-2.48; P=0.02). Postgraduate education was associated with lower odds of misuse (aOR: 0.63; 95% CI: 0.41-0.97; P=0.036).

Conclusions: Topical corticosteroid use is highly prevalent in Saudi Arabia, and nearly one-third of adults engage in at least one misuse behavior. Although overall knowledge regarding corticosteroid safety is generally satisfactory, important gaps persist regarding steroid-induced dermatoses and hidden corticosteroids in cosmetic products. Targeted educational interventions, improved patient counseling, and enhanced regulatory oversight may help reduce misuse and promote safer use of topical corticosteroids.

## Introduction

Topical corticosteroids remain among the most frequently prescribed medications in dermatology and are considered a cornerstone in the management of numerous inflammatory and immune-mediated skin disorders, including eczema, psoriasis, contact dermatitis, and other eczematous dermatoses [1-5]. Since their introduction into clinical practice, topical corticosteroids have substantially improved disease control and quality of life for millions of patients worldwide. When prescribed appropriately and used under medical supervision, these agents are generally safe and effective [3,4,6]. However, inappropriate use, prolonged application, use of excessively potent preparations, and unsupervised self-medication may result in significant adverse effects and treatment-related complications [5-7].

The widespread availability of topical corticosteroids has contributed to increasing concerns regarding misuse in many parts of the world [1,4,6]. In addition to medically indicated use, these medications are frequently employed for cosmetic purposes, including facial brightening, treatment of pigmentation disorders, and skin whitening [5,8]. Furthermore, topical corticosteroids are often incorporated into combination preparations that may be obtained without adequate medical guidance [1,6]. Such practices may expose individuals to unnecessary risks, particularly when corticosteroids are applied to sensitive anatomical sites such as the face or used for prolonged periods [3,7]. Reported adverse effects include skin atrophy, telangiectasia, acneiform eruptions, rosacea-like dermatitis, pigmentary alterations, striae, hypertrichosis, and rebound dermatitis following discontinuation. In addition, inappropriate corticosteroid use may mask or exacerbate infectious dermatoses, resulting in delayed diagnosis and more complicated clinical presentations [6-10].

Topical corticosteroid misuse has increasingly emerged as a public health concern, particularly in regions where access to these medications may occur without strict prescription control [1,4]. The growing influence of social media and online health information has further altered patterns of medication use, allowing recommendations regarding dermatologic treatments to spread rapidly beyond traditional healthcare settings [2-7]. Although digital platforms can improve public access to health information, they may also facilitate the dissemination of inaccurate or misleading advice regarding the safety and effectiveness of topical corticosteroids [1,4,8]. Consequently, individuals may initiate treatment without appropriate evaluation, continue therapy for excessive durations, or use corticosteroid-containing products without recognizing their active ingredients [3,5].

Despite increasing recognition of topical corticosteroid misuse globally, data describing public awareness, knowledge, and utilization practices in Saudi Arabia remain limited. Existing studies have generally been restricted to specific geographic regions, selected patient populations, or particular aspects of corticosteroid use [1-10]. Moreover, relatively few investigations have comprehensively evaluated the relationship between public knowledge, awareness of steroid-induced dermatoses, patterns of corticosteroid use, and factors associated with misuse at a national level. Understanding these factors is essential for developing targeted educational interventions, informing regulatory policies, and promoting safer use of topical corticosteroids within the community.

Therefore, the present study aimed to assess public awareness, knowledge, and practices regarding topical corticosteroid use and steroid-induced dermatoses among adults in Saudi Arabia. In addition, we sought to estimate the prevalence of topical corticosteroid misuse, characterize patterns of use and self-reported adverse effects, and identify demographic and behavioral factors independently associated with misuse.

## Materials and methods

Study design and setting

A national cross-sectional survey was conducted between January and March 2026 to assess public awareness, knowledge, and practices regarding topical corticosteroid use and steroid-induced dermatoses among adults in Saudi Arabia.

Study population and eligibility criteria

Adults aged 18 years or older who were residing in Saudi Arabia during the study period and were able to understand Arabic or English were eligible to participate. Individuals younger than 18 years of age and respondents who submitted incomplete questionnaires were excluded from the final analysis. To minimize duplicate responses, only one submission per participant was permitted.

Sample size calculation

The minimum required sample size was calculated using a single-population proportion formula, assuming a prevalence of topical corticosteroid misuse of 50%, a confidence level of 95%, and a margin of error of 5%. This yielded a minimum sample size of 385 participants. To improve representativeness, permit subgroup analyses, and compensate for potentially incomplete responses, the study aimed to recruit a substantially larger sample from all regions of Saudi Arabia.

Sampling strategy and data collection

A non-probability convenience sampling approach was employed. Data were collected using a structured, self-administered online questionnaire distributed through commonly used social media platforms, including WhatsApp, X (formerly Twitter), Telegram, Instagram, and Snapchat. Participants were encouraged to share the survey link within their social networks to maximize geographic coverage across the Kingdom.

**Figure 1:**
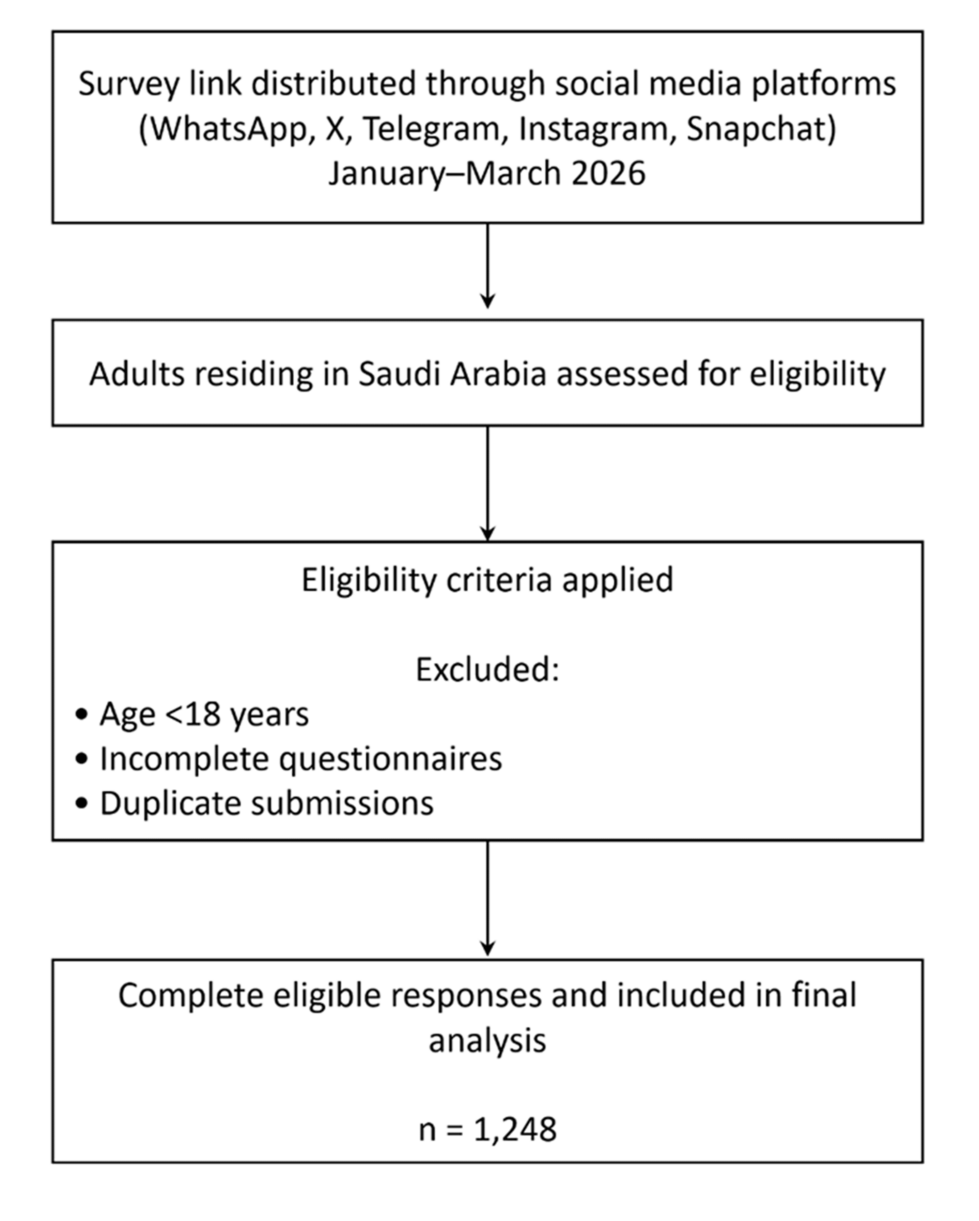
Flow diagram of participant recruitment and selection The flowchart illustrates participant recruitment, eligibility assessment, exclusions, and inclusion in the final analysis of the national cross-sectional survey evaluating topical corticosteroid use, awareness, and misuse among adults in Saudi Arabia.

Before participation, respondents were provided with an electronic information sheet describing the study objectives, voluntary nature of participation, estimated completion time, and confidentiality measures. Electronic informed consent was obtained from all participants before access to the questionnaire was granted. The participant recruitment and selection process is summarized in Figure 1.

Survey instrument

The study questionnaire was developed following a review of the dermatology and public health literature on topical corticosteroid use, misuse, and steroid-induced dermatoses. The instrument consisted of seven sections addressing demographic characteristics, history of topical corticosteroid use, patterns of use and misuse, awareness of steroid-induced dermatoses, knowledge regarding topical corticosteroids, self-reported adverse effects, and sources of information regarding skin medications.

The questionnaire included multiple-choice items, dichotomous questions, Likert-scale statements, and knowledge-based questions. Respondents who reported never using topical corticosteroids were automatically directed to the relevant sections of the survey through conditional branching logic (see Appendices).

Content validity and pilot testing

The preliminary questionnaire was reviewed by a panel of consultant dermatologists and academic faculty members with expertise in dermatology, epidemiology, and survey-based research to assess content validity, clarity, and relevance. Minor modifications were made based on expert feedback.

A pilot study involving 30 participants from the target population was subsequently conducted to evaluate comprehensibility, item interpretation, survey flow, and completion time. Data obtained during pilot testing were not included in the final analysis. Internal consistency of the knowledge and awareness domains was assessed using Cronbach's alpha, with values ≥0.70 considered indicative of acceptable reliability.

Study variables

The primary outcome was topical corticosteroid misuse. Misuse was defined a priori as the presence of at least one of the following behaviors: use without prescription, use for cosmetic purposes, use based on social media recommendations, sharing topical corticosteroids with others, prolonged unsupervised use, reuse of previous prescriptions without medical consultation, or use for fungal infections.

Secondary outcomes included knowledge regarding topical corticosteroids, awareness of steroid-induced dermatoses, patterns of corticosteroid use, and self-reported adverse effects.

Knowledge was assessed using a series of objective questions addressing indications, adverse effects, and safe use practices. Correct responses were assigned 1 point, and incorrect or "don't know" responses were assigned 0 points. A total knowledge score was subsequently calculated, with higher scores indicating greater knowledge.

Statistical analysis

Data were exported from the survey platform and analyzed using IBM SPSS Statistics for Windows, V. 29.0 (IBM Corp., Armonk, NY, USA). Categorical variables were summarized as frequencies and percentages, whereas continuous variables were summarized as means and standard deviations or medians and interquartile ranges, as appropriate. Variables demonstrating statistical significance in univariate analyses, as well as clinically relevant covariates identified a priori, were entered into a multivariable logistic regression model to identify independent predictors of topical corticosteroid misuse. Adjusted odds ratios (aORs) with corresponding 95% confidence intervals (CIs) were calculated. All statistical tests were two-sided, and a P-value of less than 0.05 was considered statistically significant.

## Results

Participant characteristics

A total of 1,248 participants were included in the final analysis. The largest age group was 25-34 years, comprising 418 participants (33.5%), followed by participants aged 18-24 years (315 participants, 25.2%). Females accounted for 736 participants (59.0%), and Saudi nationals represented 1,173 participants (94.0%). Regarding educational attainment, 786 participants (63.0%) held a bachelor's degree, and 173 (13.9%) had postgraduate qualifications, whereas 289 participants (23.2%) had a high school education or lower. Healthcare workers constituted 181 participants (14.5%) of the study population. Geographically, the Central Region contributed the largest proportion of respondents (372 participants, 29.8%), followed by the Western Region (338 participants, 27.1%) and the Eastern Region (258 participants, 20.7%). Among all respondents, 959 participants (76.9%) had attained at least a bachelor's degree, indicating a predominantly well-educated study population (Table 1).

**Table 1:** Demographic and educational characteristics of the study participants (N=1,248) Data are presented as number (%). Percentages may not sum to exactly 100% because of rounding. Healthcare workers included physicians, nurses, pharmacists, and other allied health professionals.

Characteristic		No. (%)
Total participants		1,248
Age, years	18-24	315 (25.2)
	25-34	418 (33.5)
	35-44	274 (22.0)
	45-54	157 (12.6)
	≥55	84 (6.7)
Female sex		736 (59.0)
Saudi nationality		1173 (94.0)
Education level	High school or below	289 (23.2)
	Bachelor's degree	786 (63.0)
	Postgraduate degree	173 (13.9)
Healthcare workers		181 (14.5)
Region	Central	372 (29.8)
	Western	338 (27.1)
	Eastern	258 (20.7)
	Southern	171 (13.7)
	Northern	109 (8.7)

Topical corticosteroid use and indications

Overall, 842 participants (67.5%) reported previous use of a topical corticosteroid. Among corticosteroid users, 553 participants (65.7%) obtained the medication through a physician's prescription, whereas 289 participants (34.3%) reported obtaining it without a prescription. Facial application was reported by 394 users (46.8%), and 271 users (32.2%) reported use for longer than one month. Reuse of a previous prescription without medical consultation was reported by 251 users (29.8%), while 186 users (22.1%) reported sharing topical corticosteroids with another person. Additionally, 139 users (16.5%) reported using a cream recommended through social media, 178 users (21.1%) reported cosmetic use, and 264 users (31.4%) reported purchasing corticosteroids directly from a pharmacy without a prescription (Table 2).

**Table 2:** Patterns of topical corticosteroid use among the study participants with previous corticosteroid exposure (n=842) Data are presented as number (%). Percentages were calculated using the number of participants who reported previous topical corticosteroid use (n=842) as the denominator. Responses were based on self-reported corticosteroid use practices.

Variable	No. (%)
Ever used topical corticosteroids	842 (67.5)
Obtained through a physician's prescription	553 (65.7)
Obtained without a prescription	289 (34.3)
Used on the face	394 (46.8)
Used for >1 month	271 (32.2)
Reused old prescription	251 (29.8)
Shared medication with another person	186 (22.1)
Used cream recommended through social media	139 (16.5)
Used for cosmetic purposes	178 (21.1)
Purchased directly from the pharmacy without a prescription	264 (31.4)

Among corticosteroid users, eczema was the most frequently reported indication, affecting 392 participants (46.6%), followed by skin allergy or rash in 284 participants (33.7%). Other reported indications included acne (117 participants, 13.9%), melasma or pigmentation disorders (109 participants, 12.9%), psoriasis (91 participants, 10.8%), fungal infections (84 participants, 10.0%), and skin whitening (63 participants, 7.5%). Multiple responses were permitted for this question (Table 3).

**Table 3:** Self-reported indications for topical corticosteroid use among users (n=842) Data are presented as number (%). Percentages were calculated using the number of participants who reported previous topical corticosteroid use (n = 842). Participants were allowed to select more than one indication; therefore, percentages do not total 100%.

Indication	No. (%)
Eczema	392 (46.6)
Skin allergy/rash	284 (33.7)
Psoriasis	91 (10.8)
Acne	117 (13.9)
Melasma/pigmentation	109 (12.9)
Fungal infection	84 (10.0)
Skin whitening	63 (7.5)
Other	52 (6.2)

Knowledge and awareness of topical corticosteroids

Knowledge regarding the safety and adverse effects of topical corticosteroids was generally favorable. A total of 971 participants (77.8%) correctly identified skin thinning as a potential adverse effect, 886 (71.0%) recognized that corticosteroids may worsen infections, and 933 (74.8%) were aware that prolonged facial application may lead to complications. Furthermore, 1,112 participants (89.1%) correctly indicated that medical advice should be sought before prolonged use. However, only 693 participants (55.5%) were aware that cosmetic creams may contain hidden corticosteroids. The mean overall knowledge score was 5.8±1.9 (Table 4).

**Table 4:** Knowledge regarding topical corticosteroid safety and adverse effects among the study participants (N=1,248) Data are presented as number (%) unless otherwise indicated. Percentages represent participants who correctly answered each knowledge statement. The overall knowledge score was calculated by assigning 1 point for each correct response and 0 points for incorrect or "don't know" responses. SD: standard deviation

Statement correctly answered	No. (%)
Steroids may cause skin thinning	971 (77.8)
Steroids can worsen infections	886 (71.0)
Prolonged facial use can cause complications	933 (74.8)
Medical advice is recommended before prolonged use	1,112 (89.1)
Sharing prescribed steroid creams is unsafe	827 (66.3)
Hidden steroids may be present in cosmetic creams	693 (55.5)
Overall knowledge score, mean ± SD	5.8±1.9

Regarding awareness of steroid-induced dermatoses, skin thinning was the most frequently recognized complication, identified by 973 participants (78.0%). Awareness was also relatively high for worsening fungal infections (864 participants, 69.2%) and acneiform eruptions (782 participants, 62.7%). In contrast, recognition of facial redness (711 participants, 57.0%), telangiectasia (488 participants, 39.1%), and rebound flare following discontinuation (452 participants, 36.2%) was comparatively lower (Table 5).

**Table 5:** Awareness of steroid-induced dermatoses and corticosteroid-related complications among the study participants (N=1,248) Data are presented as number (%). Percentages represent participants who correctly recognized each listed condition as a potential complication of inappropriate or prolonged topical corticosteroid use.

Complication recognized	No. (%)
Skin thinning	973 (78.0)
Acneiform eruption	782 (62.7)
Facial redness	711 (57.0)
Telangiectasia	488 (39.1)
Rebound flare after discontinuation	452 (36.2)
Worsening fungal infection	864 (69.2)

Self-reported adverse effects

Among topical corticosteroid users (n=842), 247 participants (29.3%) reported experiencing at least one adverse effect. Acneiform eruptions were the most commonly reported complication, affecting 164 users (19.5%), followed by facial erythema in 128 users (15.2%) and a burning sensation in 117 users (13.9%). Other reported adverse effects included hyperpigmentation (96 users, 11.4%), skin thinning (82 users, 9.7%), telangiectasia (54 users, 6.4%), striae (31 users, 3.7%), and hypertrichosis (28 users, 3.3%) (Table 6).

**Table 6:** Self-reported adverse effects associated with topical corticosteroid use among users (n=842) Data are presented as number (%). Percentages were calculated using the number of participants who reported previous topical corticosteroid use (n=842). Participants could report more than one adverse effect; therefore, percentages do not total 100%. Adverse effects were self-reported and were not clinically verified.

Adverse effect	No. (%)
Acneiform eruption	164 (19.5)
Facial erythema	128 (15.2)
Burning sensation	117 (13.9)
Hyperpigmentation	96 (11.4)
Skin thinning	82 (9.7)
Telangiectasia	54 (6.4)
Striae	31 (3.7)
Hypertrichosis	28 (3.3)
Any adverse effect	247 (29.3)

Prevalence and predictors of topical corticosteroid misuse

Based on the predefined study criteria, 362 participants (29.0%) met the definition of topical corticosteroid misuse. The most common misuse behaviors were non-prescription use (289 participants, 23.2%), sharing medications (186 participants, 14.9%), cosmetic use (178 participants, 14.3%), long-term unsupervised use (151 participants, 12.1%), and social media-driven use (139 participants, 11.1%). Use of topical corticosteroids for fungal infections was reported by 84 participants (6.7%) (Table 7).

**Table 7:** Prevalence and patterns of topical corticosteroid misuse among the study participants (N=1,248) Data are presented as number (%). Topical corticosteroid misuse was defined as the presence of at least one of the following behaviors: non-prescription use, cosmetic use, social media-driven use, medication sharing, prolonged unsupervised use, reuse of a previous prescription without medical consultation, or use for fungal infections. Individual misuse behaviors were not mutually exclusive.

Variable	No. (%)
Any misuse behavior	362 (29.0)
Non-prescription use	289 (23.2)
Cosmetic use	178 (14.3)
Social media-driven use	139 (11.1)
Sharing medication	186 (14.9)
Long-term unsupervised use	151 (12.1)
Use for fungal infection	84 (6.7)

In univariate analyses, misuse was significantly more common among females than males (250/736 (34.0%) vs. 112/512 (21.9%); P<0.001). Healthcare workers demonstrated lower rates of misuse compared with non-healthcare workers (34/181 (18.8%) vs. 328/1067 (30.7%); P=0.002). Participants identified as social media users exhibited substantially higher rates of misuse than non-users (139/248 (56.0%) vs. 223/1000 (22.3%); P<0.001). Similarly, participants with postgraduate education demonstrated lower misuse rates than those with lower educational attainment (34/173 (19.7%) vs. 328/1075 (30.5%); P=0.008) (Table 8).

**Table 8:** Univariate analysis of the factors associated with topical corticosteroid misuse among the study participants (N=1,248) Data are presented as number of participants with misuse/total number of participants within each subgroup (%). P-values were calculated using the chi-squared test. Statistical significance was defined as a two-sided P-value of <0.05.

Variable	Misuse, n/N (%)	P-value
Male	112/512 (21.9)	<0.001
Female	250/736 (34.0)	
Healthcare worker	34/181 (18.8)	0.002
Non-healthcare worker	328/1067 (30.7)	
Social media users	139/248 (56.0)	<0.001
Non-users	223/1000 (22.3)	
Postgraduate education	34/173 (19.7)	0.008
Lower education	328/1075 (30.5)	

Multivariable logistic regression analysis demonstrated that social media recommendation was the strongest independent predictor of misuse (aOR: 3.48; 95% CI: 2.51-4.82; P<0.001). Female sex (aOR: 1.72; 95% CI: 1.29-2.31; P<0.001), facial application of topical corticosteroids (aOR: 1.89; 95% CI: 1.40-2.55; P<0.001), and non-healthcare occupation (aOR: 1.64; 95% CI: 1.08-2.48; P=0.02) were independently associated with increased odds of misuse. In contrast, postgraduate education was independently associated with lower odds of misuse (aOR: 0.63; 95% CI: 0.41-0.97; P=0.036) (Table 9).

**Table 9:** Multivariable logistic regression analysis of the factors independently associated with topical corticosteroid misuse aOR were derived from a multivariable logistic regression model evaluating factors associated with topical corticosteroid misuse. Variables entered into the model included sex, healthcare occupation, educational attainment, social media recommendation, and facial application of topical corticosteroids. aOR: adjusted odds ratio; CI: confidence interval

Variable	aOR	95% CI	P-value
Female sex	1.72	1.29-2.31	<0.001
Social media recommendation	3.48	2.51-4.82	<0.001
Non-healthcare occupation	1.64	1.08-2.48	0.02
Facial application	1.89	1.40-2.55	<0.001
Postgraduate education	0.63	0.41-0.97	0.036

## Discussion

This national cross-sectional survey provides important insights into public awareness, knowledge, and practices regarding topical corticosteroid use in Saudi Arabia. Several noteworthy findings emerged. First, topical corticosteroid use was highly prevalent, with more than two-thirds of respondents reporting previous use. Second, nearly one-third of participants met the predefined criteria for topical corticosteroid misuse. Third, although overall knowledge regarding corticosteroid safety was generally favorable, substantial gaps remained in awareness of steroid-induced dermatoses and hidden corticosteroids in cosmetic products. Finally, social media recommendation, female sex, facial application, and non-healthcare occupation were independently associated with an increased likelihood of misuse.

The widespread use of topical corticosteroids observed in this study is not unexpected given their established role in the management of numerous inflammatory skin disorders. However, the finding that 34.3% of users obtained topical corticosteroids without a prescription and that 31.4% purchased these agents directly from pharmacies without medical supervision raises important concerns regarding inappropriate access and use. These findings support previous observations from several countries in the Middle East, South Asia, and Africa, where over-the-counter availability of topical corticosteroids has been identified as a major contributor to misuse and subsequent adverse effects [2-8]. The ease of obtaining these medications may create a perception that topical corticosteroids are inherently safe, potentially encouraging prolonged or inappropriate use.

A particularly important finding was the prevalence of misuse, affecting 29.0% of participants. Although the definition of misuse varies across studies, this proportion suggests that inappropriate corticosteroid practices remain common within the Saudi population. The most frequently reported behaviors included non-prescription use, medication sharing, cosmetic use, and prolonged unsupervised application. These practices are clinically significant because they increase the risk of both local and systemic adverse effects while potentially delaying appropriate diagnosis and treatment of underlying dermatologic conditions [3,10].

The use of topical corticosteroids for cosmetic purposes warrants particular attention. More than one-fifth of corticosteroid users reported cosmetic use, and a notable proportion reported use for pigmentation disorders or skin-whitening purposes. This observation reflects a broader phenomenon reported in multiple regions worldwide, where corticosteroid-containing products are promoted for skin lightening, facial brightening, or rapid improvement of cosmetic concerns. Such use is problematic because many individuals may be unaware of the presence of corticosteroids in combination products or may underestimate the risks associated with prolonged facial application. The relatively low awareness observed in our study regarding hidden corticosteroids in cosmetic preparations further supports this concern [2-8].

Despite generally favorable knowledge scores, important deficiencies in awareness persisted. While most respondents recognized skin thinning, worsening infections, and facial complications as potential adverse effects, fewer participants were aware of telangiectasia and rebound dermatitis following corticosteroid discontinuation. These findings suggest that public understanding is largely focused on commonly discussed adverse effects, whereas recognition of more specific manifestations of steroid-induced dermatoses remains limited. Such knowledge gaps may contribute to delayed recognition of complications and continued inappropriate use [10-14].

The pattern of self-reported adverse effects observed in the present study is consistent with the known safety profile of topical corticosteroids. Nearly one-third of users reported at least one adverse effect, with acneiform eruptions, facial erythema, and a burning sensation being the most frequently reported manifestations. These findings are clinically plausible given that facial application was reported by almost half of corticosteroid users and emerged as an independent predictor of misuse in multivariable analysis. The predominance of facial adverse effects highlights the particular vulnerability of facial skin to corticosteroid-induced complications and reinforces existing recommendations for cautious use in this anatomical region.

One of the most striking findings of this study was the strong association between social media exposure and misuse. Participants identified as social media users demonstrated markedly higher rates of misuse, and social media recommendations remained the strongest independent predictor of inappropriate corticosteroid use after adjustment for potential confounders. This finding has important implications in the current digital era, where health information is increasingly obtained from online platforms. Although social media can facilitate the dissemination of health education, it may also contribute to the spread of inaccurate information regarding dermatologic treatments. The influence of non-medical recommendations may be particularly relevant for cosmetic concerns, where anecdotal testimonials and unregulated advertising can shape consumer behavior.

Female participants were also more likely to engage in misuse behaviors. Although the underlying reasons cannot be fully determined from the present study, this association may reflect greater utilization of topical products for cosmetic purposes, heightened concern regarding pigmentation disorders, or increased exposure to beauty-related content on social media platforms. Similarly, healthcare workers and individuals with postgraduate education demonstrated lower rates of misuse, suggesting that health literacy and educational attainment may offer a degree of protection against inappropriate corticosteroid practices.

The findings of this study have several implications for clinical practice and public health policy. Dermatologists, primary care physicians, and pharmacists should actively counsel patients regarding the risks associated with unsupervised corticosteroid use, particularly when treatment involves the face or prolonged duration. Educational campaigns aimed at improving public awareness of steroid-induced dermatoses may help reduce misuse and facilitate earlier recognition of adverse effects. Furthermore, stricter enforcement of regulations governing the dispensing of topical corticosteroids may reduce non-prescription access and limit inappropriate use. Given the strong influence of social media identified in this study, future educational initiatives should incorporate digital platforms and collaborate with trusted healthcare professionals to provide accurate dermatologic information.

This study has several strengths. It included a large sample drawn from multiple geographic regions of Saudi Arabia, allowing the assessment of topical corticosteroid use across a diverse population. The survey instrument comprehensively evaluated knowledge, awareness, practices, adverse effects, and predictors of misuse, enabling a multidimensional assessment of corticosteroid-related behaviors. In addition, multivariable regression analysis allowed the identification of independent factors associated with misuse while accounting for potential confounding variables.

Several limitations should also be considered. First, the cross-sectional design precludes the establishment of causal relationships between identified predictors and misuse behaviors. Second, data were self-reported and therefore subject to recall bias and social desirability bias. Third, the online recruitment strategy may have favored participation by younger and more educated individuals, potentially limiting generalizability to populations with lower internet access. Fourth, adverse effects were not clinically verified and may have been over- or under-reported by participants. Finally, although the study achieved broad geographic representation, the use of convenience sampling may introduce selection bias.

## Conclusions

In this national cross-sectional survey, topical corticosteroid use was highly prevalent among adults in Saudi Arabia, and nearly one-third of participants demonstrated at least one form of misuse. Although overall knowledge regarding topical corticosteroid safety was generally satisfactory, important gaps remained in awareness of steroid-induced dermatoses and hidden corticosteroids in cosmetic products. Non-prescription access, cosmetic use, medication sharing, and social media-driven recommendations were common contributors to inappropriate use, while social media influence emerged as the strongest independent predictor of misuse. These findings highlight the need for targeted public education initiatives, enhanced patient counseling by healthcare professionals, and strengthened regulatory oversight of topical corticosteroid dispensing. Improving awareness of the risks associated with unsupervised corticosteroid use may help reduce preventable adverse effects and promote safer, evidence-based use of these commonly utilized dermatologic therapies.

## Appendices

**Table 10:** Survey questionnaire used to assess awareness, knowledge, and misuse of topical corticosteroids Questions marked with an asterisk (*) permitted multiple responses. Participants who answered "No" to Question 7 were directed to Sections 5-7 through conditional branching logic. The questionnaire was administered electronically in Arabic and English.

Section	Item no.	Survey question	Response options
Participant information	1	Age	18-24; 25-34; 35-44; 45-54; ≥55
	2	Gender	Male; Female; Prefer not to say
	3	Nationality	Saudi; Non-Saudi
	4	Region of residence	Central; Western; Eastern; Northern; Southern
	5	Highest educational level	Primary school; Intermediate school; High school; Bachelor's degree; Postgraduate degree
	6	Occupation	Healthcare worker; Student; Government employee; Private sector employee; Unemployed; Other
History of topical corticosteroid use	7	Have you ever used a topical corticosteroid cream/ointment?	Yes; No; Not sure
	8	Who recommended or prescribed it?	Dermatologist; Non-dermatologist physician; Pharmacist; Family/friend; Social media/internet; Self-decision; Other
	9	For what condition did you use it?*	Eczema; Psoriasis; Skin allergy/rash; Acne; Facial pigmentation/melasma; Skin whitening; Fungal infection; Unknown; Other
	10	Which area did you apply it to?*	Face; Neck; Hands; Body; Groin/armpits; Multiple areas
	11	Duration of use	Less than 1 week; 1-4 weeks; 1-3 months; 3-6 months; More than 6 months
	12	Did you use the medication exactly as instructed?	Yes; No; Partially
	13	Have you ever used a steroid cream without a medical prescription?	Yes; No
	14	Have you ever shared a steroid cream with another person?	Yes; No
	15	Have you ever reused an old steroid prescription without consulting a doctor?	Yes; No
Practices and misuse	16	Have you ever used a steroid cream for cosmetic purposes?	Yes; No
	17	If yes, for what purpose?*	Skin whitening; Brightening/glow; Acne treatment; Dark spots; Melasma; Other
	18	Have you ever purchased a steroid cream directly from a pharmacy without a prescription?	Yes; No
	19	Have you ever used a cream recommended through social media?	Yes; No
	20	Do you usually read the medication leaflet before use?	Always; Sometimes; Never
	21	Do you check whether a cream contains steroids before using it?	Always; Sometimes; Never
	22	Have you ever used a combination cream containing steroids without knowing it contained steroids?	Yes; No; Not sure
Awareness of steroid-induced dermatoses	23	Long-term use of topical steroids can cause skin thinning	Strongly agree; Agree; Neutral; Disagree; Strongly disagree
	24	Topical steroids can worsen fungal infections if used improperly	Strongly agree; Agree; Neutral; Disagree; Strongly disagree
	25	Using topical steroids on the face should generally be supervised by a physician	Strongly agree; Agree; Neutral; Disagree; Strongly disagree
	26	Topical steroids can cause acne-like eruptions	Strongly agree; Agree; Neutral; Disagree; Strongly disagree
	27	Excessive use of topical steroids can cause facial redness	Strongly agree; Agree; Neutral; Disagree; Strongly disagree
	28	Topical steroids may lead to visible blood vessels (telangiectasia)	Strongly agree; Agree; Neutral; Disagree; Strongly disagree
	29	Not all skin creams are safe for long-term use	Strongly agree; Agree; Neutral; Disagree; Strongly disagree
	30	Strong steroid creams are not necessarily better than mild ones	Strongly agree; Agree; Neutral; Disagree; Strongly disagree
	31	Children are more vulnerable to side effects from topical steroids	Strongly agree; Agree; Neutral; Disagree; Strongly disagree
	32	Sudden discontinuation after prolonged facial steroid use may cause rebound symptoms	Strongly agree; Agree; Neutral; Disagree; Strongly disagree
	33	Misuse of steroid creams is a public health issue in Saudi Arabia	Strongly agree; Agree; Neutral; Disagree; Strongly disagree
Knowledge assessment	34	Topical steroids can be used indefinitely without side effects	True; False; Don't know
	35	Steroid creams are effective for every type of skin rash	True; False; Don't know
	36	Steroid creams may cause skin thinning	True; False; Don't know
	37	Steroid creams can worsen some infections	True; False; Don't know
	38	Prolonged use on the face may lead to complications	True; False; Don't know
	39	Medical advice should be sought before long-term use	True; False; Don't know
	40	It is safe to use someone else's prescribed steroid cream	True; False; Don't know
	41	Skin-lightening creams may contain hidden corticosteroids	True; False; Don't know
Personal experience with adverse effects	42	Have you ever experienced any side effects after using a steroid cream?	Yes; No; Not sure
	43	If yes, which side effects did you experience?*	Acne; Facial redness; Burning sensation; Increased pigmentation; Skin thinning; Stretch marks; Increased hair growth; Visible blood vessels; Recurrent fungal infection; Other
	44	Did you seek medical advice for these side effects?	Yes; No
	45	Were you diagnosed by a physician with a steroid-induced skin condition?	Yes; No; Not sure
Sources of information	46	Where do you obtain information about skin creams?*	Dermatologist; Other physician; Pharmacist; Family/friends; Social media; Internet websites; Television/radio
	47	Which source do you trust the most?	Dermatologist; Pharmacist; Physician; Social media; Internet websites; Family/friends
	48	Would you like more public education regarding safe use of topical steroid creams?	Yes; No; Not sure

## References

[1] Akhter F, Araf S, Hossain MF, Haque MA, Islam MR (2025). The dangers of misuse of corticosteroid drugs in treating superficial fungal infections: presentation of a case series for stricter policy regulation. Public Health Chall.

[2] Coondoo A (2014). Topical corticosteroid misuse: the Indian scenario. Indian J Dermatol.

[3] Dey VK (2014). Misuse of topical corticosteroids: a clinical study of adverse effects. Indian Dermatol Online J.

[4] Lahiri K, Coondoo A (2016). Topical steroid damaged/dependent face (TSDF): an entity of cutaneous pharmacodependence. Indian J Dermatol.

[5] Lu H, Xiao T, Lu B, Dong D, Yu D, Wei H, Chen HD (2010). Facial corticosteroid addictive dermatitis in Guiyang City, China. Clin Exp Dermatol.

[6] Mahar S, Mahajan K, Agarwal S, Kar HK, Bhattacharya SK (2016). Topical corticosteroid misuse: the scenario in patients attending a tertiary care hospital in New Delhi. J Clin Diagn Res.

[7] Miller JA, Munro DD (1980). Topical corticosteroids: clinical pharmacology and therapeutic use. Drugs.

[8] Nicol NH, Baumeister LL (1997). Topical corticosteroid therapy. Considerations for prescribing and use. Lippincotts Prim Care Pract.

[9] Saraswat A, Lahiri K, Chatterjee M (2011). Topical corticosteroid abuse on the face: a prospective, multicenter study of dermatology outpatients. Indian J Dermatol Venereol Leprol.

[10] Sharma R, Abrol S, Wani M (2017). Misuse of topical corticosteroids on facial skin. A study of 200 patients. J Dermatol Case Rep.

[11] Shrestha S, Joshi S, Bhandari S (2020). Prevalence of misuse of topical corticosteroid among dermatology outpatients. JNMA J Nepal Med Assoc.

[12] Srivastava A (2019). A clinicoepidemiological study of topical corticosteroid misuse at a tertiary care center. J Dermatolog Treat.

[13] Verma SB (2015). Topical corticosteroid misuse in India is harmful and out of control. BMJ.

[14] Young M (2025). Topical corticosteroid use in everyday clinical practice: cautionary tales. J Clin Aesthet Dermatol.

